# IgG4-related pleural disease in a patient with pulmonary adenocarcinoma under durvalumab treatment: a case report

**DOI:** 10.1186/s12890-020-1150-x

**Published:** 2020-04-25

**Authors:** Takeshi Terashima, Eri Iwami, Takashi Shimada, Aoi Kuroda, Tatsu Matsuzaki, Takahiro Nakajima, Aya Sasaki, Keisuke Eguchi

**Affiliations:** 1 Department of Respiratory Medicine, Tokyo Dental College Ichikawa General Hospital, 5-11-13 Sugano, Ichikawa, Chiba, 272-0824 Japan; 2 Department of Pathology and Laboratory Medicine, Tokyo Dental College Ichikawa General Hospital, 5-11-13 Sugano, Ichikawa, Chiba, 272-0824 Japan; 3 Department of Surgery, Tokyo Dental College Ichikawa General Hospital, 5-11-13 Sugano, Ichikawa, Chiba, 272-0824 Japan

**Keywords:** Durvalumab, IgG4-related pleural disease, Immune checkpoint inhibitors, Immune-related adverse events, Lung cancer

## Abstract

**Background:**

Immune checkpoint inhibitors (ICIs) are the standard treatment for non-small cell lung cancer. The unique adverse events that can arise after treatment with ICIs are known as immune-related adverse events (irAE). As the number of cases under treatment with ICIs increases, new types of characteristics of irAE have emerged. This case report suggests that IgG4-related pleural disease could occur as an irAE.

**Case presentation:**

A 64-year-old man was diagnosed with pulmonary adenocarcinoma stage IIIB. Following concurrent chemoradiotherapy, durvalumab was administered every two weeks. The patient complained of dyspnea on effort 4 months after the initiation of durvalumab therapy. Chest CT scans showed mild bilateral pleural effusion 4 months after the initiation of durvalumab therapy, and the amount of pleural effusion increased further at 7 months. Durvalumab was thought to be a potential cause of pleural effusion and was withdrawn after 13 courses of administration over 7 months. The level of serum IgG4 was 2750 mg/dL. The levels of IgG4 of the pleural fluids were 2790 mg/dL on the right side and 2890 mg/dL on the left side at 7 months. Microscopic examination of the pleural biopsy revealed lymphoplasmacytic infiltration with storiform fibrosis. Immunohistochemical examinations showed that the number of IgG4-positive cells was > 20/high power field and the percentage of IgG4-positive to IgG-positive plasma cells was > 50%. Oral prednisolone at a dose of 30 mg/day was initiated, and remarkable clinical improvements were achieved. After 4 months of prednisolone therapy, the level of serum IgG4 decreased to 370 mg/dL and chest CT revealed the disappearance of bilateral pleural effusion.

**Conclusion:**

This was a case of IgG4-related pleural disease in a patient with pulmonary adenocarcinoma under durvalumab treatment. To our knowledge, this is the first case report of IgG4-related pleural disease as an irAE. It is important to consider the possibility of IgG4-related pleural disease in cases of pleural effusion during the treatment with ICIs.

## Background

Immune checkpoint inhibitors (ICIs) are currently the standard treatment for non-small cell lung cancer (NSCLC). Recently, the administration of a programmed death-ligand 1 (PD-L1) inhibitor following concurrent chemoradiotherapy (CRT) for stage III NSCLC has demonstrated a longer 24-month overall survival of 66.3% than that of 55.6% for controls [1]. The unique adverse events that can arise after treatment with ICIs, including pneumonitis, colitis, and thyroiditis, are known as immune-related adverse events (irAE) [2]. As the number of cases under treatment with ICIs increases, new characteristics of irAE have been emerged. However, IgG4-related pleural disease has never been reported as irAE.

Herein, we report a case of IgG4-related pleural disease in a patient with pulmonary adenocarcinoma under durvalumab treatment. To our knowledge, this is the first case report of IgG4-related pleural disease as irAE.

## Case presentation

A 64-year-old man was diagnosed with pulmonary adenocarcinoma stage IIIB. The patient had a smoking history of 50 packs a year. Immunohistochemical analysis revealed a PD-L1 tumor proportion score of > 50%. Concurrent CRT using weekly courses of carboplatin plus paclitaxel and 60.0 Gy irradiation over 6 weeks was performed and partial response was achieved. Consolidation immunotherapy was initiated 4 weeks after the CRT treatment concluded. Durvalumab (10 mg/kg), an ICI, was administered every 2 weeks. The patient complained of dyspnea on effort 4 months after the initiation of durvalumab therapy.

**Fig. 1:**
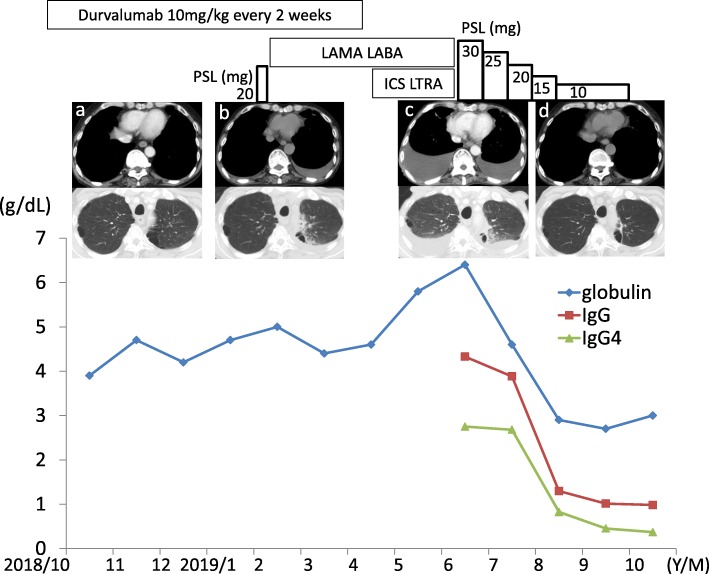
Clinical course. Chest computed tomography showed the appearance of mild bilateral pleural effusion 4 months after the initiation of durvalumab (**b**). Durvalumab was withdrawn after 13 courses of administration over 7 months. There were increases in the amount of bilateral pleural effusion (**c**), serum levels of globulin, IgG, and IgG4 8 months after the initiation of durvalumab. PSL, at a dose of 30 mg per day, was started and gradually decreased. PSL therapy for 4 months decreased the level of IgG4 from 2750 mg/dL to 370 mg/dL and induced complete disappearance of bilateral pleural effusion (**d**). LAMA: long-acting muscarinic antagonist; LABA: long-acting beta-agonist; ICS: inhaled corticosteroid; LTRA; leukotriene receptor antagonist; PSL: prednisolone

Chest X-ray and computed tomography (CT) scans showed radiation pneumonitis in the irradiated area accompanied with mild bilateral pleural effusion (Fig. 1b). There were no signs of the progression of lung cancer or thromboembolism. Because pleural effusion was relatively small, the performance of pleurocentesis proved technically difficult. Spirometry revealed a volume capacity of 1.77 L, a forced expiratory volume in 1 s (FEV1) of 1.01 L, %FEV1 of 39.2%, and FEV1/forced volume capacity of 61.0%, suggesting severe obstructive disease by chronic obstructive pulmonary disease (COPD) combined with constrictive disease by radiation pneumonitis. Laboratory examination revealed eosinophilia (1165 /μL) and serum IgE level of 2000 IU/mL, suggesting an asthma and COPD overlap. The echocardiogram showed normal cardiac function and pulmonary artery pressure. The cause of the dyspnea was assumed to be exacerbation of asthma and COPD overlap.

The patient was treated with a long-acting muscarinic antagonist, a long-acting beta-agonist, a moderate dose of inhaled corticosteroid, a leukotriene receptor antagonist, and a diuretic. The patient was also administered systemic oral corticosteroid (prednisolone 20 mg/day) for five days and antibiotics (levofloxacin) for seven days 4 months after the initiation of durvalumab therapy. Despite these treatments, the dyspnea was progressive. The chest X-ray and CT scans showed increases in the amounts of bilateral pleural effusion (Fig. 1c). No progression of radiation pneumonitis or lung cancer was observed in either lung field. Durvalumab was withdrawn after 13 courses of administration over 7 months because there was a possibility that the pleural effusion was induced by durvalumab. The patient was admitted for the treatment of pleural effusion.

Physical examination did not show any findings suggesting the presence of collagen vascular diseases. The levels of C-reactive protein and angiotensin-converting enzyme were 0.13 mg/dL (normal range < 0.15 mg/dL) and 8.6 IU/mL (normal range 7.7–29.4 IU/mL), respectively. The T-SPOT test for TB, which measures the number of interferon-gamma-secreting spot-forming T cells obtained from a patient stimulated by *Mycobacterium tuberculosis*-specific antigens, was negative. There was an increase in the level of serum immunoglobulin of 6.4 g/dL. The level of serum IgG was 4.329 g/dL (normal range 0.87–1.7 g/dL), and among the IgG subclass, IgG4 was 2.750 g/dL (normal range 0.0045–0.117 g/dL). Serologic studies indicated that an anti-nuclear antibody was positive. Serum myeloperoxidase- anti-neutrophil cytoplasmic antigen was negative (< 1.0 U/mL). The levels of thyroid stimulating hormone, free triiodothyronine, and free thyroxine were 2.92 μIU/mL (normal range 0.54–4.26 μIU/mL), 2.12 pg/mL (normal range 2.39–4.06 pg/mL), 0.87 ng/dL (normal range 0.71–1.52 ng/dL), respectively.

Pleurocentesis was performed on each side separately and revealed unilateral exudative pleural effusion with a predominance of mononuclear cells with no malignant cells. Bacterial culture and polymerase chain reaction analysis of the pleural fluids for *Mycobacterium tuberculosis*, *avium*, or *intacellulare* DNA were all negative. Adenosine deaminase concentrations were 47.2 U/L and 49.3 U/L in the right- and left-sided pleural fluids, respectively. The levels of IgG and IgG4 of the pleural fluids were 4183 mg/dL and 2790 mg/dL on the right side, and 4366 mg/dl and 2890 mg/dL on the left side.

**Fig. 2:**
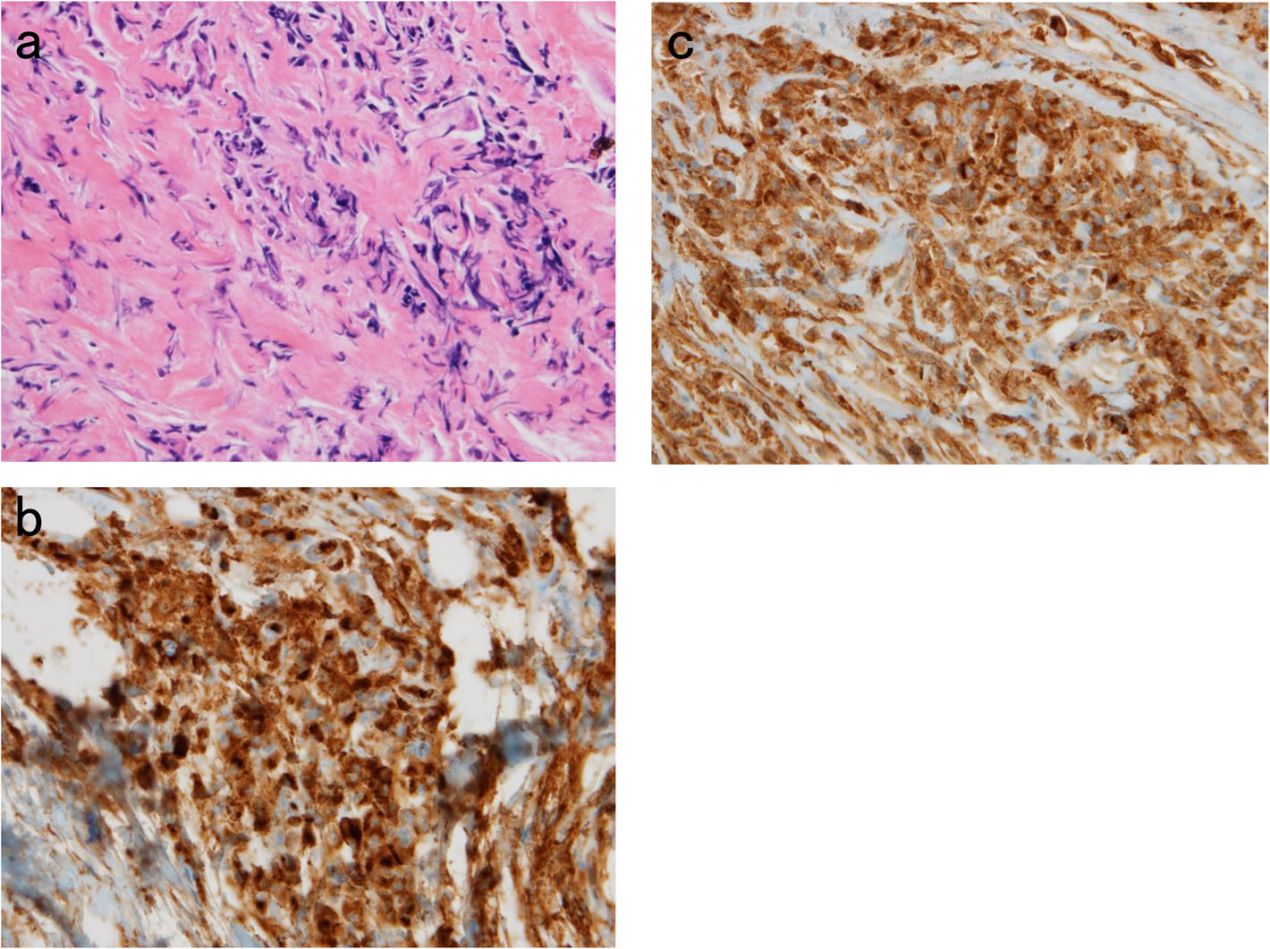
**(a)** Microscopic examination revealed lymphoplasmacytic infiltration with storiform fibrosis. **(b)** Immunochemical staining showed the presence of numerous IgG4-positive plasma cells. The number of IgG4-positive cells was > 20/high power field (× 400). **(c)** Immunochemical staining showed the presence of IgG-positive plasma cells (× 400)

On the 12th day of hospitalization, a pleural biopsy was performed using video-associated thoracoscopy and the specimen was collected from the pleura on the right side. Microscopic examination revealed lymphoplasmacytic infiltration with storiform fibrosis (Fig. 2a). There was no evidence of granulomas, necrosis, or malignancy. Immunohistochemical examinations showed the presence of numerous IgG4-positive plasma cells. The number of IgG4-positive cells was > 20/high power field (× 400) (Fig. 2b) and the percentage of IgG4-positive to IgG-positive plasma cells (Fig. 2c) was > 50%. These findings indicated that IgG4-related disease contributed to the pathogenesis of pleural effusion.

Oral prednisolone at a dose of 30 mg/day was initiated and remarkable clinical improvements were achieved. After 4 months of prednisolone therapy, chest CT scans revealed the complete disappearance of bilateral pleural effusion (Fig. 1d), the level of serum IgG4 was decreased to 0.37 g/dL (Fig. 1), and the dyspnea was resolved. Presently, the patient is under treatment with an oral corticosteroid and under careful observation for the recurrence of adenocarcinoma.

## Discussion and conclusions

This is a rare case of IgG4-related respiratory and pleural diseases in a patient with pulmonary adenocarcinoma under treatment with an ICI, durvalumab. Known irAEs that can arise after treatment with ICI include: pneumonitis, colitis, and thyroiditis [2]. However, there have been no reports describing IgG4-related pleural disease as irAE [2, 3].

The criteria of IgG4-related respiratory disease include an abnormal shadow on chest CT, serum level of IgG4 higher than 135 mg/dL and characteristic findings in tissue specimens [4–6]. In the present case, two pieces of evidence suggested the contribution of IgG4-related respiratory disease to the pleural effusion: 1. extremely high concentration of IgG4 in the serum and 2. the concentrations of IgG4 in the bilateral pleural effusion that were higher than that of the serum. This assumption was further confirmed by the marked IgG4-positive plasma cell infiltration with characteristic pattern of fibrosis in the pleural biopsy specimen. Differential diagnoses of IgG4-related respiratory diseases in the present case included malignant lymphoma, multicentric Castleman’s disease, collagen vascular diseases, and sarcoidosis [5, 6]. The finding that there were no increases in the levels of C-reactive protein, angiotensin-converting enzyme, and anti-neutrophil cytoplasmic antigen suggests that it is unlikely that these diseases were the cause of pleural effusion in the present case.

Among the eight extant cases describing IgG4-related pleural disease, three cases reported the levels of IgG4 in the pleural effusion to be 124 to 653 mg/dL, and in all eight cases, the levels of serum IgG4 were 136 to 740 mg/dL. Clinical responses to corticosteroid therapy were observed in these cases [7]. According to another report describing the clinicopathological features of five cases of IgG4-related pleural disease, the average age was 62 years, and two patients had no organ involvement other than pleural involvement [8].

IgG4 itself is considered a non-inflammatory immunoglobulin and the actual role of IgG4 itself in the process of IgG4-related disease remains unclear. T helper 2 cells, CD4+ cytotoxic T cells, and T follicular helper cells are among the T helper subsets that are thought to be the drivers of the pathogenesis of IgG4-related diseases. Inflammatory cytokines produced by activation of these T helper cells contribute to the expansion of IgG4-producing plasma cells and activation of fibroblasts. CD4+ cytotoxic T cells and T follicular helper cells play key roles in the production of IgG4 by plasmablasts and plasma cells [9]. Durvalumab, which is a PD-L1 inhibitor, upregulates functions of T cells by inhibiting the programmed cell death protein 1 (PD-1)/PD-L1 pathway [10]. T cells, antibodies, and cytokine responses may be involved in the pathogenesis of irAE [2]. The PD-1/PD-L1 pathway also contributes to the interaction between T follicular helper cells and plasmablasts [9]. One possible mechanism is that the inhibition of PD-1/PD-L1 pathway enhances T follicular helper cells to promote the growth, differentiation, and class switching of B cells to IgG4 [11, 12]. Another possibility is that there is an association between pulmonary adenocarcinoma and IgG4-related disease. IgG4-related diseases have been known to have a high incidence of malignancies [13]. However, the latter theory is unlikely because pulmonary adenocarcinoma was followed by IgG4-related pleural disease in the present case.

When our patient who had a history of heavy smoking complained of dyspnea during ICI treatment after CRT for lung cancer, the differential diagnoses were exacerbation of COPD, cardiac failure, pulmonary thromboembolism, progression of the lung cancer, and radiation pneumonitis. ICI-related adverse events, such as interstitial pneumonitis were also excluded. Differential diagnoses for pleural effusion included pleuritis carcinomatosa, radiation-induced pleuritis, tuberculosis, cardiac failure, renal failure, and hypothyroidism [14]. This case report is novel and relevant because it demonstrates that IgG4-related respiratory and pleural diseases should be considered as a differential diagnosis in patients undergoing ICI with complicating pleural effusion. In the present case, the increase in the level of serum immunoglobulin was positively associated with the increase in the amount of pleural effusion. In such a case, measurement of the levels of IgG4 in the serum and pleural effusion is recommended.

IgG4-related disease is frequently accompanied by bronchial asthma [9, 15]. In the present case, the presence of COPD was previously known based on the heavy smoking history and the findings of pulmonary function test and chest CT. The level of serum IgE had not measured prior to durvalumab treatment. The eosinophilia and the high level of serum IgE emerged following therapy with durvalumab. Eosinophilia and increased levels of serum IgE, both reported in 40% of patients with IgG4-related disease, are mediated by inflammatory cytokines released from T helper cells [9, 15]. It is possible that eosinophilia and the increase in the level of serum IgE were also induced by ICI as well as IgG-related pleural disease.

To the best of our knowledge, this is the first report of IgG4-related respiratory and pleural disease in a patient with adenocarcinoma during treatment of ICI. It is important to take into consideration the IgG4-related pleural disease in a case of pleural effusion during the treatment with ICIs.

## Abbreviations

CRT: chemoradiotherapy
COPD: chronic obstructive pulmonary disease
CT: computed tomography
FEV1: forced expiratory volume in 1 s
ICI: immune-checkpoint inhibitor
irAE: immune-related adverse events
NSCLC: non-small cell lung cancer
PD-1: programmed cell death protein 1
PD-L1: programmed death-ligand 1.
